# Pregnancy in a Previously Conjoined Thoracopagus Twin with a Crisscross Heart

**DOI:** 10.1155/2015/594537

**Published:** 2015-07-27

**Authors:** Bassam H. Rimawi, Iris Krishna, Anurag Sahu, Martina L. Badell

**Affiliations:** ^1^Division of Maternal-Fetal Medicine, Department of Gynecology and Obstetrics, Emory University School of Medicine, 8th Floor, 550 Peachtree Street, Atlanta, GA 30308, USA; ^2^Department of Congenital Cardiology, Emory University School of Medicine, 8th Floor, 550 Peachtree Street, Atlanta, GA 30308, USA

## Abstract

*Background*. Crisscross heart (CCH) is a complex, rare, congenital, rotational, cardiac abnormality that accounts for <0.1% of congenital heart defects (CHD). CCH is characterized by the crossing of the inflow streams of the two ventricles due to an abnormal twisting of the heart. A case of maternal CCH has not been previously reported. *Case*. We report a case of a primigravida with a CCH, who was separated at birth from her thoracopagus conjoined twin. Pregnancy was managed by congenital cardiology, maternal-fetal medicine, anesthesiology, and obstetrics. She underwent a 39-week vaginal delivery without maternal or neonatal complication. *Conclusion*. A successful term pregnancy outcome was achieved in a patient with CCH using a multidisciplinary approach to address her cardiac condition.

## 1. Introduction

The incidence of multiple gestations continues to increase, now accounting for more than 3% of all live births in the United States [1]. Conjoined twins are the rarest subset of monochorionic gestations, occurring in approximately 1 in 50,000 to 1 in 190,000 births [2]. The majority of conjoined twins do not survive and their outcome depends on the type of fusion and associated defects [2]. Conjoined twins are classified according to the most prominent shared anatomy. Thoracopagus classification accounts for 75% of conjoined twins and describes fetuses that have a common sternum, diaphragm, upper abdominal wall, liver, pericardium, and gastrointestinal tract [2]. Most of the thoracopagus twins share cardiac anatomy and/or a congenital heart defect (CHD) is present in one or both twins [2].

Crisscross heart (CCH) is a complex, rare, congenital, rotational abnormality that accounts for <0.1% of congenital heart defects (CHD) [3]. This CHD is characterized by the crossing of the inflow streams of the two ventricles due to an apparent twisting of the heart about its long axis. In general, neonates born with CCH have an overall poor outcome with cyanosis commonly noted at birth. Depending on the severity of other cardiac anomalies that are commonly associated with CCH, such as transposition of the great arteries, double outlet right ventricle, pulmonary stenosis, and atrioventricular defects, symptoms of heart failure can be also encountered at birth [3]. Pregnancy among patients with CHD is associated with maternal and fetal complications.

A literature search did not reveal any prior cases of pregnancy in women with CCH; we herein present a case of a primigravida with CCH separated at birth from her thoracopagus twin who underwent successful term vaginal delivery without complication.

## 2. Case Report

A 29-year-old gravida 1, para 0, with a history as a thoracopagus conjoined twin presented to our prenatal clinic at 15 weeks of gestation. She underwent a successful separation from her sister at 10 months of age. Her cotwin has no medical problems with respect to her history as a conjoined twin and specifically no cardiac complications. Conversely, our patient's cardiac anatomy was complicated by a CCH, situs solitus with dextrocardia, and a secundum-type atrial septal defect (ASD). At time of separation, she underwent a fenestrated ASD closure. At age 3, she was diagnosed with Ebstein's anomaly and at the age of 25 she underwent tricuspid valve replacement with a 33 mm Edwards bovine valve. Since that surgery, she had been doing well walking approximately 5 miles a day and followed closely by her adult congenital cardiologist who recommended against pregnancy given the unknown risk of maternal morbidity with CCH.

Despite the regular use of combined oral contraceptive pills, she became pregnant and was dated by a second trimester ultrasound at 15 weeks. She and her husband were counseled about the potential risks of pregnancy with respect to her CCH and tricuspid valve replacement. At 20 weeks, detailed anatomy scan and formal fetal echo were performed which were both normal.

Echocardiographic assessment performed during this pregnancy illustrated situs solitus with dextrocardia. Our patient had typical features of CCH: the right-sided right atrium connects with the left-sided (posterior) right ventricle and the left-sided left atrium connects to the right-sided (anterior) left ventricle. She also had normal ventriculoarterial concordance. Systemic LV function was in the normal range and the mean gradient across the tissue tricuspid valve prostheses was 10 mmHg.

A multidisciplinary team that involved adult congenital cardiology, maternal-fetal medicine, primary obstetrics, and anesthesiology followed her pregnancy closely. Despite the complexity of her diagnosis, the patient remained symptom-free and had an uneventful antenatal course with the exception of well-controlled A1 gestational diabetes. During her prenatal care, her congenital cardiologist, as well as her maternal-fetal medicine obstetrician, carefully monitored her closely, and a maternal echocardiogram was performed one week prior to her delivery and was stable when compared to her previous echocardiograms. She appeared stable, and, after a lengthy discussion between her obstetrician and cardiologists, an induction of labor with vaginal delivery was felt to be most appropriate, with cesarean delivery reserved for other maternal or fetal indications. An elective induction of labor was recommended at 39 weeks of gestation. She underwent an uncomplicated induction of labor via Foley balloon and pitocin with an epidural for regional anesthesia. She delivered via normal vaginal delivery a 2920-gram male infant with APGAR score of 9 and 9 at 1 and 5 minutes, respectively. During her postpartum course, she required oral torsemide for mild volume overload. She and her baby were discharged home on postpartum day four.

## 3. Comment

Conjoined twins are the rarest subset of monozygotic twins occurring as a result of incomplete zygotic division that occurs 13 to 15 days after fertilization. In general, 25–30% will die prior to delivery, 40–50% immediately after birth, and 15–20% surviving thereafter [4]. There are no trials available regarding the most appropriate management of these affected pregnancies and termination is usually recommended. In particular, the presence of thoracopagus conjoined twins as this type is frequently accompanied by shared cardiac anatomy and prognosis for surgical division is very poor [4].

CCH is a rare, complex, congenital, rotational abnormality that is also referred to as superoinferior ventricles. This rare condition occurs when the systemic and pulmonary venous streams cross at the atrioventricular (AV) level without mixing. The exact incidence of this congenital heart condition is unknown; however, in general, it accounts for less than eight per one million and less than 0.1% of all congenital heart defects [5]. The AV structures of a normal heart are parallel to each other when viewed from the front of the heart. In contrast with CCH, the AV structures are not parallel but angulated by as much as 90 degrees [6]. Another feature of a CCH is the connection of the atrium with the contralateral ventricle, as well as the ventricular chambers being arranged in a superoinferior fashion, with the right ventricle (RV) superiorly and the left ventricle (LV) inferiorly located, regardless of whether the AV connection is concordant or discordant [7]. Our patient had a CCH with a concordant AV connection. Of note, most patients with CCH have other associated congenital cardiac anomalies, such as a transposition of the great vessels, right ventricular hypoplasia, ventricular septal defects, subpulmonary stenosis, and straddling AV valve. Our patient had an Ebstein's anomaly with an associated secundum-type atrial septal defect (ASD) in addition to CCH, for which she underwent surgical repair. Based on the WHO risk stratification for pregnancy in women with CHD, given that she had a repaired heart defect with no residual lesions, she would theoretically be considered a low risk candidate, with an overall mortality of <1% [8]. However, the WHO risk score does not take into full account this entire patient's cardiovascular risk; therefore, she is at moderate risk for adverse pregnancy complications. The angulation of the bioprosthetic valve related to CCH leading to impairment in right ventricular inflow and the small right ventricle and risk of arrhythmia in patients with Ebstein's do not fit into any typical risk algorithm. Therefore her degree of risk was somewhat uncertain.

Conjoined twins and CCH are very rare amongst themselves, and the combination of both occurring in a pregnant mother has not been previously described in the literature. Amongst the other described rare congenital heart defects in pregnant women, successful pregnancies have occurred in patients with a single ventricle and transposition of the great arteries [4]. As compared to those case reports, a successful pregnancy was achieved in this case without any major complications.

The presence of the bioprosthetic tricuspid valve in relation to her CCH was of particular concern in our patient. The valve was seated abnormally from its typical position due to the angulation associated with CCH. As such, the gradient across the valve was increased despite the leaflets themselves being of normal function. This along with a right ventricle that was smaller than usual due to her history of Ebstein's was a significant concern as to how her right ventricle would tolerate the volume load of pregnancy. Fortunately, her right ventricle tolerated the pregnancy well and she only required a few doses of oral diuretics postpartum.

The desire for a patient with a complex congenital heart defect to become and/or continue a pregnancy should be evaluated on a case-by-case basis taking into consideration their baseline functional status and anticipated response to pregnancy changes based on their underlying pathophysiology. The maternal/perinatal risks should be reviewed with these patients using the best information we have. A multidisciplinary team is required in the pregnancy planning and management. This team approach requires active participation of maternal-fetal medicine specialists, adult congenial heart specialists, obstetricians, and anesthesiologists with experience in managing the pregnant women with congenital heart disease.
